# Chronic irradiation with 222-nm UVC light induces neither DNA damage nor epidermal lesions in mouse skin, even at high doses

**DOI:** 10.1371/journal.pone.0201259

**Published:** 2018-07-25

**Authors:** Kouji Narita, Krisana Asano, Yukihiro Morimoto, Tatsushi Igarashi, Akio Nakane

**Affiliations:** 1 Department of Microbiology and Immunology, Hirosaki University Graduate School of Medicine, Hirosaki, Aomori, Japan; 2 Institute for Animal Experimentation, Hirosaki University Graduate School of Medicine, Hirosaki, Aomori, Japan; 3 Department of Biopolymer and Health Science, Hirosaki University Graduate School of Medicine, Hirosaki, Aomori, Japan; 4 Ushio Inc., Chiyoda-ku, Tokyo, Japan; Georgetown University, UNITED STATES

## Abstract

Surgical site infections (SSIs) represent an important clinical problem associated with increased levels of surgical morbidity and mortality. UVC irradiation during surgery has been considered to represent a possible strategy to prevent the development of SSI. 254-nm UVC induces marked levels of DNA damage by generating cyclobutyl pyrimidine dimers (CPD) in microorganisms. However, this effect is elicited not only in microorganisms, but also in human cells, and chronic exposure to 254-nm UVC has been established to represent a human health hazard. In contrast, despite short wavelength-UVC light, especially 222-nm UVC, having been demonstrated to elicit a bactericidal effect, single irradiation with a high dose of 222-nm UVC energy has been reported to not induce mutagenic or cytotoxic DNA lesions in mammalian cells. However, the effect of chronic irradiation with a high dose of 222-nm UVC to mammalian cells has not been determined. In this study, it was demonstrated that large numbers of CPD-expressing cells were induced in the epidermis of mice following treatment with a small amount of single exposure 254-nm UVC, and then less than half of these cells reduced within 24 h. Chronic 254-nm UVC irradiation was revealed to induce sunburn and desquamation in mouse skin. Histological analysis demonstrated that small numbers of CPD-expressing cells were detected only in hyperkeratotic stratum corneum after chronic irradiation with a high dose of 254-nm UVC, and that significant hyperplasia and intercellular edema were also induced in the epidermis of mice. In contrast, chronic irradiation with 222-nm UVC light was revealed not to induce mutagenic or cytotoxic effects in the epidermis of mice. These results indicated that 222-nm UVC light emitted from the lamp apparatus (or device), which was designed to attenuate harmful light present in wavelengths of more than 230 nm, represents a promising tool for the reduction of SSI incidence in patients and hospital staff.

## Introduction

Surgical site infections (SSIs) are defined as infections that affect either the incision or deep tissue at operation sites, typically occur within 30 days of an operative procedure and are associated with increased levels of surgical morbidity and mortality. Despite improvements in preventive treatment, including antisepsis of hands and forearms, sterilization of surgical instruments and preparation of the patient’s skin, SSIs remain a significant clinical problem [1, 2].

Microbiota present on the patient’s skin and mucous membranes have been well established to represent an important pathogen source of SSIs [3]. On the other hand, it has also been reported that approximately 80–90% of bacterial contaminants found in wounds post-surgery are due to air in the operating room. Hence, the reduction of airborne bacterial contaminants may represent an effective strategy for decreasing the incidence of SSIs [4].

It has been well established that UVC light within the range of 240–280 nm elicits a significant germicidal effect [5]. Germicidal lamps with an emission peak at 254-nm have been widely used to kill and inactivate bacteria and viruses [6]. It has previously been reported that UVC irradiation produced by a germicidal lamp reduced the number of environmental bacteria in an operating room and lowered the risk of wound infection following joint replacement surgery performed in the operating room [7]. Furthermore, it has been established that 254-nm UVC is directly absorbed by DNA in microorganisms and induces a variety of mutagenic and cytotoxic DNA lesions [8]. Cyclobutyl pyrimidine dimers (CPD) caused by UV radiation interrupt transcription, translation and replication of DNA leads to bacterial cell death and viral inactivation [8]. It has been previously reported that chronic UV irradiation induces skin damage. Chronic UVB irradiation induces a large number of CPD in mouse epidermal cells. However, the number of cells retaining CPD decreased within several days and following irradiation with UVB light, epidermal hyperplasia subsequently occurred in skin [9]. The induction of tumorigenesis following chronic irradiation with a high dose of UVB light to mouse skin was reported to be dependent on the accumulation of CPD as well as the degree of epidermal hyperplasia [10]. Similarly, treatment with 254-nm UVC also induces CPD accumulation in mammalian cells and epidermal hyperplasia, and repeated exposure to 254-nm UVC for long durations of time is known to represent a human health hazard, cause dermatitis and increase the risk of skin cancer [8, 11]. These studies demonstrated that when UVC irradiation is applied for the prophylaxis of SSIs, use of personal protective equipment is necessary for patients and hospital staff in order to prevent 254-nm UVC exposure during surgical procedures [12].

UVC light with decreased wavelengths (~200–230 nm) has been reported to be harmless to mammalian cells since it does not reach the nuclei, as the light is absorbed by proteins, particularly by peptide bonds and other biomolecules [13, 14, 15]. It has been reported that irradiation with 222-nm UVC light efficiently reduces bacterial counts of MRSA *in vitro* without inducing mouse skin damage, which is typically induced by conventional germicidal UV exposure *in vivo* [14]. We previously demonstrated that single irradiation with 222-nm UVC light reduces the number of bacterial MRSA in mouse skin wounds immediately after the irradiation, and does not induce CPD in mouse epidermal cells [16]. These studies demonstrated that single irradiation with 222-nm UVC may be harmless to the epidermis of mice. Treatment with 222-nm UVC irradiation in operating rooms during surgery may represent an effective strategy for the prophylaxis of SSIs without eliciting harmful effects to patients and hospital staff. However, the effect of chronic 222-nm UVC irradiation to mammalian skin has not yet been determined.

In this study, it was demonstrated that single irradiation with 254-nm UVC induced CPD-retaining cells in the epidermis, which then decreased to almost half of the initial value a total of 24 h after irradiation. Following the treatment of mice with chronic irradiation using a high dose of 254-nm UVC light for 10 days, a small number of CPD-retaining cells were detected in the hyperkeratotic stratum corneum alone. Conversely, significant hyperplasia and intercellular edema were induced in the epidermis of mice following chronic irradiation. In contrast, chronic irradiation with a high dose of 222-nm UVC did not induce the CPD-retaining cells or epidermal abnormalities in mouse dorsal skin.

## Materials and methods

### Mice and ethics statement

Seven-week-old female hairless mice (Hos: HR-1; Japan SLC, Inc., Hamamatsu, Japan) were used in this study. Mice were maintained under specific pathogen-free conditions at the Institute for Animal Experimentation, Hirosaki University Graduate School of Medicine. All experiments were carried out in strict accordance with the Guidelines for Animal Experimentation of Hirosaki University. The experimental protocol was approved by the ethics committee of the Institute for Animal Experimentation, Hirosaki University Graduate School of Medicine (Permit number: M16010).

### UVC light source

**Fig 1:**
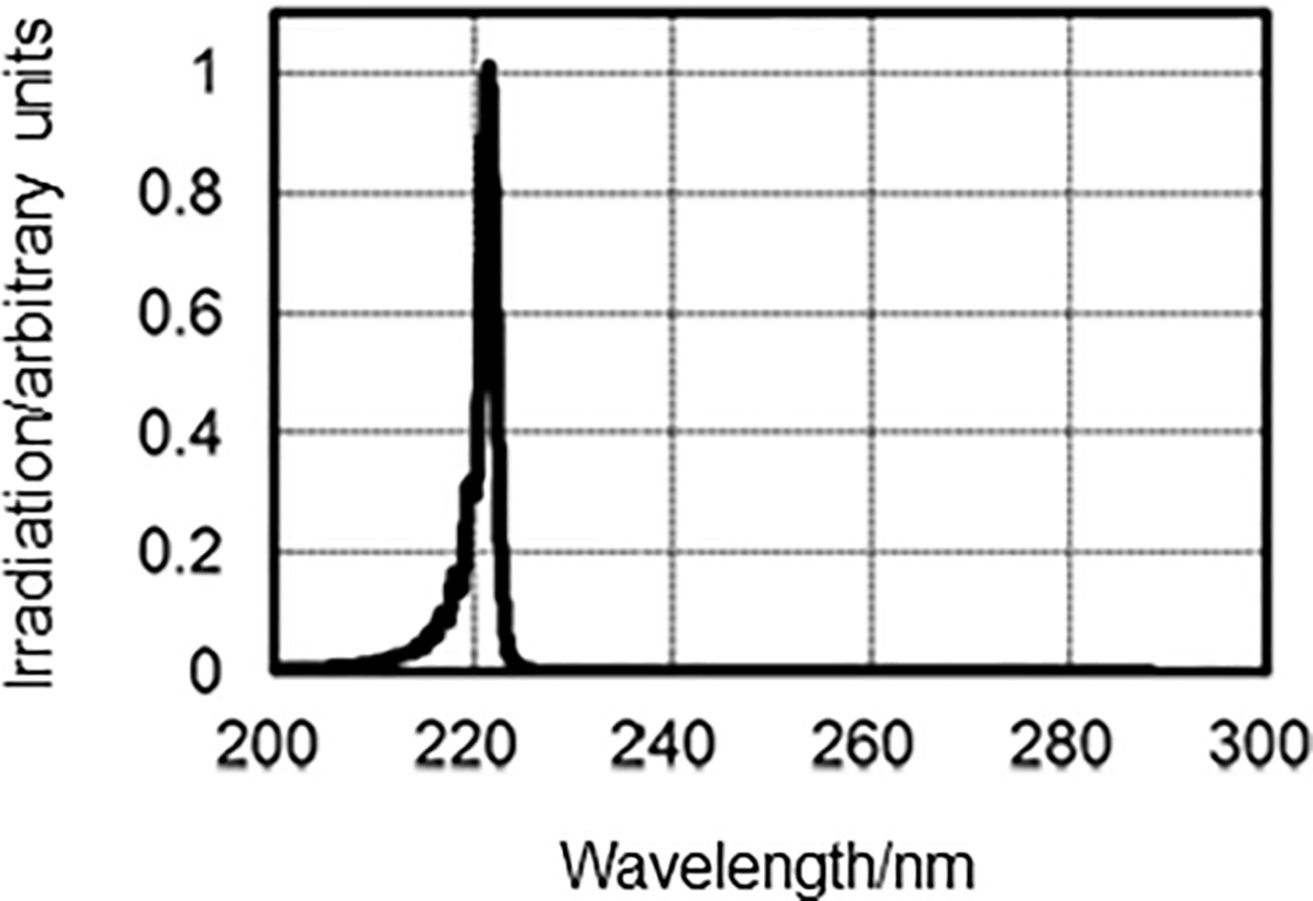
Measured spectra emitted from the Kr-Cl excimer lamp equipped with a band-pass filter.

Two types of lamp devices were used for UVC light irradiation: one was mounted using a krypton-chloride (Kr-Cl) excimer lamp and an optical filter that restricted spectra emitting light ranging between 200-230-nm, of which the maximum output wavelength was 222-nm; and the other was a conventional low-pressure mercury lamp named SUV-4 (AS ONE corp. Osaka, Japan), which emitted a line spectrum at 254-nm. The 222-nm-emitting SafeZoneUVC device (Ushio Inc. Tokyo, Japan) is composed of a lamp, air-cooling fan, mirrors and a custom band-pass filter. The filter was used to block almost all wavelengths except for the dominant 222-nm emission wavelength (Fig 1). Irradiance emitted by 222-nm light was measured using an S-172/UIT250 accumulated UV meter (Ushio Inc.), and was found to be 5 mW/cm^2^ at a distance of 10 mm from the emission window. Irradiance emitted by 254-nm light was determined by an S-254/UIT250 (Ushio Inc.), and was revealed to be 3 mW/cm^2^ at a distance of 20 mm from the window.

### UVC irradiation

Two experiments were performed in this study: One to observe the recovery rate of CPD generated by irradiation subjected to the dorsal skin of hairless mice using a small amount of 254 nm-light, and the other to evaluate the toxicity associated with chronic irradiation. Groups of five mice were anesthetized with isoflurane (Pfizer Japan Inc., Tokyo, Japan), and their dorsal skins were then irradiated using 254-nm UVC at 75 mJ/cm^2^. We previously reported that mice subjected to single irradiation treatment of 222-nm at 450 mJ/cm^2^ exhibited significantly reduced MRSA bacterial counts on dorsal skin [16]. To evaluate the effect of chronic 254 or 222-nm UVC irradiation on dorsal skin, mice were anesthetized with isoflurane (Pfizer Japan Inc.) and subsequently subjected to daily irradiation with 254-nm or 222-nm UVC light at a dose of 450 mJ/cm^2^/day on days 1, 2, 3, 4, 5, 8, 9 and 10. Gross appearances of irradiated skin were observed immediately after irradiation.

### Histological and immunohistochemical analysis

The dorsal skins of mice were immediately harvested at 1, 3, 6 and 24 h time intervals post-irradiation with 254-nm UVC at 75 mJ/cm^2^. In addition, the dorsal skins of mice subjected to irradiation with UVC at 450 mJ/cm^2^ were collected 1 day after the last treatment. Skin tissues were then fixed with 10% phosphate-buffered formalin overnight. Paraffin-embedded tissue and 2 μm-thick tissue sections were prepared. After deparaffinization and rehydration of the tissue sections, hematoxylin and eosin staining was performed. To detect CPD formation, skin sections were prepared as described above, and antigen retrieval was performed by incubating the sections in proteinase K solution (1:1000; Qiagen, Hilden, Germany). The sections were then incubated with HRP-conjugated anti-CPD monoclonal antibodies (Kaniya Biomedical Co., Seattle, WA) overnight at 4°C. After rinsing three times in PBS for 5 min each, the color reaction was developed by addition of diaminobenzidine. Following this, counterstaining was performed with hematoxylin. CPD-positive cells were quantified by counting the cells in 10 random visual fields of each section (200× magnification).

## Results

### Time course of DNA damage repair following single 254-nm UVC irradiation

**Fig 2:**
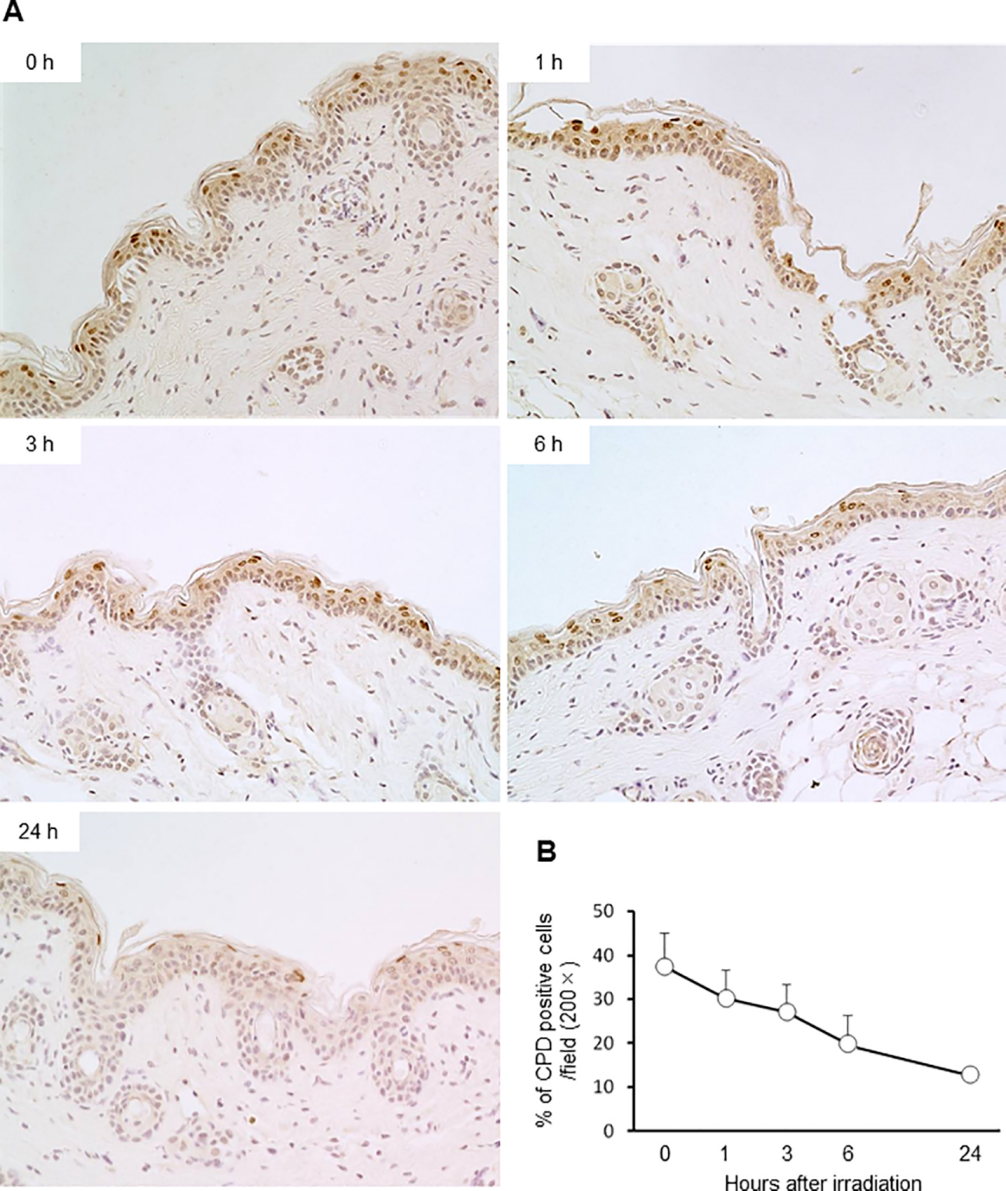
Time-course analysis of DNA damage repair following 254-nm UVC irradiation. Dorsal skins of mice irradiated with 254-nm UVC at 75 mJ/cm^2^ were immediately collected post-treatment, as well as 1, 3, 6 and 24 h post-irradiation. (A) CPD-expressing cells were detected by immunohistochemistry (200× magnification). (B) Percentages of CPD-expressing cells per visual field (200× magnification) were enumerated (n = 5).

To investigate DNA repair following CPD expression induced by single irradiation with 254-nm UVC, dorsal skin of hairless mice was irradiated with 254-nm UVC light at 75 mJ/cm^2^. The results revealed that CPD-expressing cells were distributed in the stratum spinosum immediately after irradiation. Furthermore, the distribution of CPD-expressing cells at 1 h after irradiation was similar to that observed immediately after irradiation. CPD-expressing cells were then detected in the upper stratum spinosum at 3 and 6 h after irradiation. A total of 24 h after irradiation, CPD-expressing cells appeared flattened in shape and were only detected on surface of the epidermis (Fig 2A). In addition, 37% of keratinocytes expressed CPD immediately after irradiation, and the number of CPD-expressing cells then gradually reduced to 13% a total of 24 h post-irradiation (Fig 2B).

### Effect of chronic irradiation with 222-nm UVC on epidermis of mouse skin

**Fig 3:**
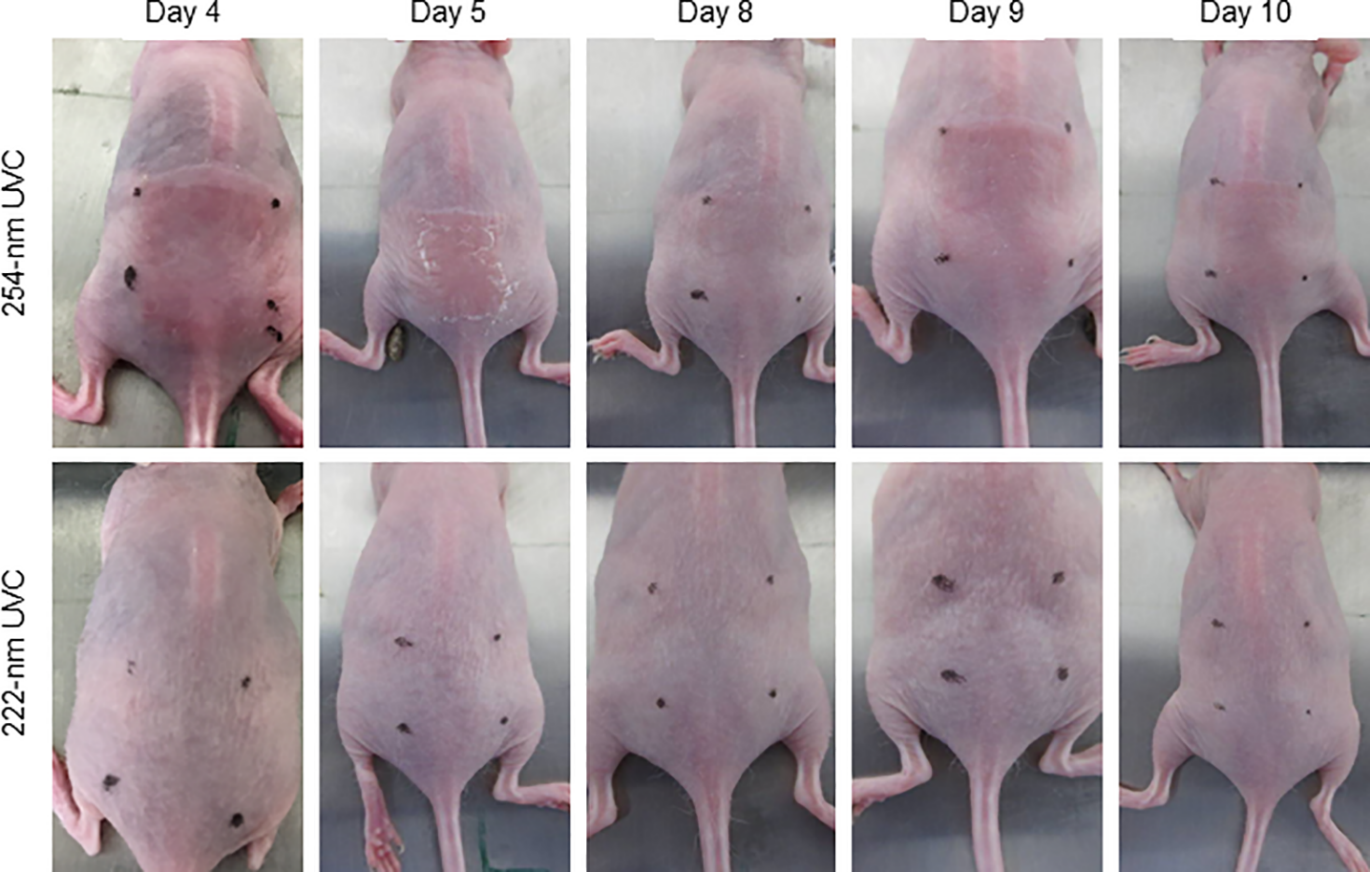
Gross appearance of dorsal skin of mice intermittently irradiated with 254-nm or 222-nm UVC. Dorsal skins of mice subjected to daily irradiation with 254-nm or 222-nm UVC at 450 mJ/cm^2^/day for a total of 10 days. The gross appearance of the irradiated skin specimens was immediately evaluated after irradiation.

**Fig 4:**
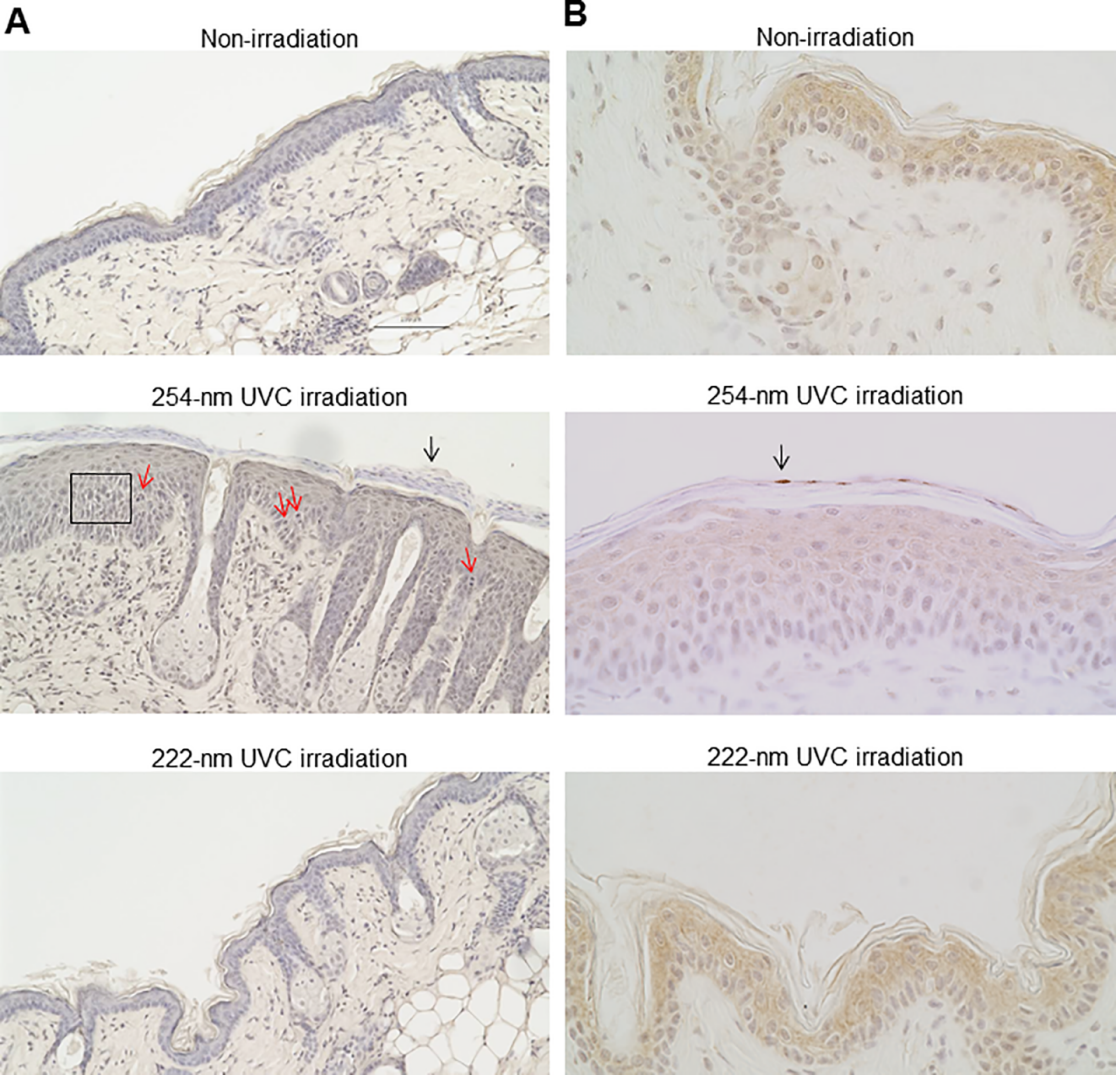
Histological analysis of dorsal skin of mice intermittently irradiated with 254-nm or 222-nm UVC. The dorsal skin of mice was subjected to daily sham-irradiation or daily irradiation with 254-nm or 222-nm UVC at 450 mJ/cm^2^/day for a total of 10 days. At 1 day post-termination of treatment, skin specimens were collected and stained with hematoxylin and eosin (x200 magnification). (A) Epidermal hyperplasia, intracellular edema (square), mitotic figures (red arrow) in the stratum spinosum epidermidis and hyperkeratosis (black arrow) were detected on the dorsal skin of mice irradiated with 254-nm UVC. (B) Skin sections were stained with anti-CPD antibodies (x400 magnification). Arrows indicate CPD-expressing cells.

To investigate epidermal damage induced by chronic irradiation using 254-nm and 222-nm UVC light, mice were irradiated a total of 8 times according to the aforementioned protocol. As shown in Fig 3, sunburn and desquamation were observed in the dorsal skins of mice irradiated with 254-nm UVC, not with 222-nm UVC, on days 4 and 5. Although sunburn and desquamation symptoms were found to be attenuated on day 8, the abnormal findings were exacerbated in the 254-nm UVC-irradiated mice on days 9 and 10. In the dorsal skin of 222-nm irradiated mice as well as non-irradiated mice, these abnormal findings were not observed during evaluation (Fig 3). To further investigate the effect of chronic irradiation with 222-nm UVC and 254-nm UVC on the epidermis of mouse skin, histological analyses were performed. The dorsal skins of mice that had been irradiated 8 times with 254-nm UVC exhibited parakeratosis, epidermal hyperplasia, intracellular edema and mitotic figures in the stratum spinosum. These histological findings were not observed in the epidermis of mice irradiated with 222-nm UVC or in non-irradiated mice (Fig 4A). In contrast, CPD-expressing cells in the skin of mice subjected to chronic irradiation with 254-nm UVC were detected only in the hyperkeratotic stratum corneum, but not in the stratum spinosum. CPD-expressing cells were not detected in the epidermis of mice irradiated with 222-nm UVC or in non-irradiated mice (Fig 4B).

## Discussion

UVC irradiation emitted by germicidal lamps in operation rooms during surgery has been considered to represent a potential strategy for the reduction of SSIs, as airborne bacteria have been revealed to be closely associated with the incidence of SSIs [7, 12, 17]. It has been established that the mechanism of using 254-nm UVC light for bactericidal effects impairs the genetic materials in which CPD is predominantly damaged in bacterial DNAs [6, 18]. However, exposure of human cells to 254-nm UVC causes the formation of mutagenic and cytotoxic DNA lesions, which, if exposed to for a sufficient duration of time, may lead to the initiation and progression of skin cancer [11]. Alternatively, mammalian cells have numerous repair systems for UV-induced DNA lesions. Nucleotide excision repair (NER) is one of the most versatile and flexible repair systems [19]. It has previously been reported that approximately 30% of CPD-retaining human epidermal keratinocytes are repaired within 24 h post-UVB irradiation *in vitro* [20]. In addition, elimination of cells damaged with UVC exposure by apoptosis is considered to be a protective function against skin cancer [21]. Effect of chronic 222-nm UVC irradiation to apoptosis including via p53-dependent intrinsic and CD95-dependent extrinsic pathways is not filly elucidated. In the present study, the number of CPD-retaining cells in epidermis, which received single irradiation with 254-nm UVC, gradually reduced to less than half of the preliminary total within 24 h (Fig 2B). It has been reported that epidermal basal cells retain the ability to continuously proliferate, daughter cells migrate toward the surface of the skin and that the estimated epidermal turnover period of mice is 8–10 days [22]. Buonanno *et al*. reported that 254-nm UVC irradiation induced epidermal hyperplasia in mouse skin and increased the percentage of epidermal cells expressing the proliferative marker Ki-67 by double compared with mice that had not been subjected to irradiation [11]. Fig 2A showed that single irradiation with 254-nm UVC induced polygonal CPD-expressing cells in the epidermis, excluding the basal layer, immediately after irradiation. At 3 and 6 h time intervals post-irradiation, CPD-expressing cells were revealed to be flattened and were detected in the upper layer of epidermis; after a total of 24 h post-irradiation, CPD-expressing cells were detected only in the stratum granulosum. These results suggested that decreased levels of CPD-expressing cells in the epidermis of mice within 24 h post-irradiation was due to not only repair DNA lesions by the NER system, but also the promotion of epidermal turnover induced by UVC irradiation.

It has been previously shown that 222 nm-UCV light displays comparable bactericidal properties with 254-nm UVC. *S*. *aureus* is known as the most common microbial cause of SSIs, accounting for 15–20% of nosocomial SSIs [23, 24]. At present, methicillin-resistant *S*. *aureus* (MRSA) represents a major problem worldwide. We reported that irradiation with 222 nm-UCV reduced the MRSA burden on the skin surface, as well as in the skin wounds, of mice immediately after irradiation [16]. Ponnaiya *et al*. demonstrated that 222 nm-UVC light efficiently prevented MRSA infection in a hairless mouse model of superficial skin incisions [12].

222-nm UVC light may effectively penetrate bacterial cells (diameter of <1 μm); however, 222-nm UVC light struggles to reach mammalian nuclei, as this UVC light is strongly absorbed by proteins and other biomolecules in cells (diameter ranging approximately between 10 and 25 μm) and markedly attenuated before reaching the nucleus [14, 25]. It has also been established that 222-nm UVC light fails to penetrate stratum corneum, and thus does not reach epidermal cells underlining stratum corneum [14]. Furthermore, single irradiation with 222-nm UVC has been reported to elicit a bactericidal effect in mammalian cells without inducing DNA lesions [14, 16, 12]. However, the effect of chronic irradiation with 222-nm UVC to mammalian cells remains unknown.

In the present study, irradiation with 254-nm UVC light for 5 consecutive days induced sunburn and desquamation in the epidermis of mice. Despite these effects being attenuated after a further 2 days of treatment, affected skin lesions were exacerbated following re-irradiation with 254-nm UVC for a further 3 consecutive days. In contrast, lesions were not observed in the dorsal skin of mice consecutively irradiated with 222-nm UVC, or mice that were not subjected to irradiation, during observation. Previous studies have reported that single irradiation with 254-nm UVC induces the expression of Ki-67 in a large number of cells, which is strongly associated with cell proliferation, significant epidermal hyperplasia and pre-mutagenic DNA lesions [11, 14, 26]. In this study, CPD-expressing cells were found to be distributed only in the hyperkeratotic stratum corneum; whereas significant epidermal hyperplasia, intercellular edema in the stratum spinosum and parakeratosis were observed in the epidermis of mice subjected to chronic 254-nm UVC irradiation. Berton *et al*. suggested that the etiological role of chronic UVB irradiation in tumorigenesis is strongly correlated with epidermal hyperplasia, rather than the amount of DNA photodamage [10]. Sterenborg *et al*. reported that chronic 254-nm irradiation induces hyperkeratosis, and scaly tumors are produced in hyperkeratotic areas [27]. It may be suggested that the induction of epidermal hyperplasia represents the harmful effect of chronic irradiation with 254-UVC light. Importantly, these histological lesions were not detected in the epidermis of mice subjected to chronic irradiation with 222-nm UVC mice.

In the present study, we have demonstrated that chronic irradiation with 222-nm UVC light did not induce a mutagenic or cytotoxic effect on the epidermis of mice. However, in order to perform direct application of chronic 222-nm UVC light to human skin, further investigation is required. The 222-nm UVC-emitting lamp represents a promising tool for the reduction of SSI incidence in patients and hospital staff.
